# A Racially Unbiased, Machine Learning Approach to Prediction of Mortality: Algorithm Development Study

**DOI:** 10.2196/22400

**Published:** 2020-10-22

**Authors:** Angier Allen, Samson Mataraso, Anna Siefkas, Hoyt Burdick, Gregory Braden, R Phillip Dellinger, Andrea McCoy, Emily Pellegrini, Jana Hoffman, Abigail Green-Saxena, Gina Barnes, Jacob Calvert, Ritankar Das

**Affiliations:** 1 Dascena, Inc San Francisco, CA United States; 2 Cabell Huntington Hospital Huntington, WV United States; 3 Marshall University School of Medicine Huntington, WV United States; 4 Kidney Care and Transplant Associates of New England Springfield, MA United States; 5 Division of Critical Care Medicine Cooper University Hospital/Cooper Medical School of Rowan University Camden, NJ United States; 6 Cape Regional Medical Center Cape May Court House, NJ United States

**Keywords:** machine learning, health disparities, racial disparities, mortality, prediction

## Abstract

**Background:**

Racial disparities in health care are well documented in the United States. As machine learning methods become more common in health care settings, it is important to ensure that these methods do not contribute to racial disparities through biased predictions or differential accuracy across racial groups.

**Objective:**

The goal of the research was to assess a machine learning algorithm intentionally developed to minimize bias in in-hospital mortality predictions between white and nonwhite patient groups.

**Methods:**

Bias was minimized through preprocessing of algorithm training data. We performed a retrospective analysis of electronic health record data from patients admitted to the intensive care unit (ICU) at a large academic health center between 2001 and 2012, drawing data from the Medical Information Mart for Intensive Care–III database. Patients were included if they had at least 10 hours of available measurements after ICU admission, had at least one of every measurement used for model prediction, and had recorded race/ethnicity data. Bias was assessed through the equal opportunity difference. Model performance in terms of bias and accuracy was compared with the Modified Early Warning Score (MEWS), the Simplified Acute Physiology Score II (SAPS II), and the Acute Physiologic Assessment and Chronic Health Evaluation (APACHE).

**Results:**

The machine learning algorithm was found to be more accurate than all comparators, with a higher sensitivity, specificity, and area under the receiver operating characteristic. The machine learning algorithm was found to be unbiased (equal opportunity difference 0.016, *P*=.20). APACHE was also found to be unbiased (equal opportunity difference 0.019, *P*=.11), while SAPS II and MEWS were found to have significant bias (equal opportunity difference 0.038, *P*=.006 and equal opportunity difference 0.074, *P*<.001, respectively).

**Conclusions:**

This study indicates there may be significant racial bias in commonly used severity scoring systems and that machine learning algorithms may reduce bias while improving on the accuracy of these methods.

## Introduction

Health care disparities are well documented in the United States [1]. These disparities affect the accessibility of care, quality of care, and health outcomes of racial minority groups [1-4]. Causes of these inequities are multifaceted and include socioeconomic factors, institutionalized racism, and a historically motivated lack of trust between minority populations and health care providers [1,5,6].

Technology can play a powerful role toward the effort of both exposing and minimizing disparities in health care. In particular, artificial intelligence (AI) and machine learning approaches have the potential to either maintain or reduce systemic inequities in health care settings and outcomes. Much attention has been given to the fact that AI and machine learning systems trained on data that reflects racial disparities will in turn learn and perpetuate such disparities and their influence on the health care system [7]. Several studies have found evidence that machine learning–based algorithms commonly used in health care settings exhibit differential accuracy by race [8,9]. A recent study by Vyas et al [10] found that algorithms used across a broad range of specialties, including cardiology, urology, and oncology, may exhibit differential accuracy across race even after so-called race corrections. By attempting to correct for race, these tools may in fact make it more difficult for nonwhite patients to receive appropriate care. For example, the authors note that these corrections move black patients systematically toward lower risk scores when computing cardiac mortality risk [11] and estimated kidney function [12], while deeming nonwhite patients higher risks for complications for procedures such as vaginal birth following a cesarean delivery [13] and certain cardiac surgeries [14]. Vyas et al [10] conclude that the use of these race-corrected tools may not only impact the quality and timeliness of care that nonwhite patients receive but may also enshrine certain racial disparities as fact, making disparities more difficult to minimize.

Despite the potential for bias found in specialized scoring systems, insufficient attention has yet been paid to how early warning scores and mortality scores intended for the general patient population may similarly perpetuate racial disparities in health outcomes. Many studies on the development and validation of scoring systems such as the Modified Early Warning Score (MEWS) [15] report findings from predominantly white patient samples [16] or do not report race data at all [15,17,18]. Literature directly examining the potential for racial bias in these scoring systems has found evidence of differential performance by race. Several studies of the emergency severity index (ESI) [19] have found systematic underestimation of acuity scores for nonwhite patients in general [20], pediatric [21], and veteran populations [22] when controlling for a wide range of important confounders. Similarly, a study of MEWS performance in an Asian population found reduced accuracy as compared with validation studies performed on predominantly white samples [23]. These findings have wide ranging implications and suggest the use of such scores may accentuate health disparities wherever they are used. Pressingly, their use in triaging patients during the COVID-19 crisis may contribute to disparities in COVID-19 outcomes.

To address this issue, we have developed a machine learning algorithm for the prediction of patient mortality [24], designed to minimize the potential for racial bias in algorithm prediction scores. We compare this algorithm performance to commonly used patient severity scoring systems, including MEWS, the Simplified Acute Physiology Score II (SAPS II) [25], and the Acute Physiologic Assessment and Chronic Health Evaluation (APACHE) [26] score across white and nonwhite racial groups. This study aims to determine whether a machine learning algorithm can minimize racial bias in patient risk predictions as compared with commonly used rules-based methods.

## Methods

### Data Processing

**Figure 1:**
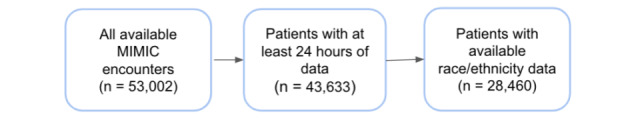
Attrition diagram for patient inclusion.

Data were drawn from the Medical Information Mart for Intensive Care–III (MIMIC-III) database [27]. The database consists of data on more than 53,000 patient encounters for patients admitted to the intensive care unit at a large academic health center between 2001 and 2012. Patients were included if they had at least 10 hours of available measurements after intensive care unit (ICU) admission, had at least one of every measurement used for model prediction, and had recorded race/ethnicity data. Patients for whom race/ethnicity was missing or recorded as declined to state or unknown were considered to have no available race/ethnicity data. Patient inclusion is shown in Figure 1. In assessing the potential for differential performance across racial groups, patients were grouped as non-Hispanic white or nonwhite.

Data were extracted on age and 13 commonly used patient measurements, including diastolic blood pressure, systolic blood pressure, heart rate, temperature, respiratory rate, oxygen saturation, white blood cell, platelets, creatinine, Glasgow coma scale, fraction of inspired oxygen, and potassium and sodium levels. Data on each measure were gathered hourly for 10 hours, beginning at the time of ICU admission. If multiple values of a single measure were recorded during a given hour, their average was taken and used. Not all measures were available for all patients. Outliers in the data, defined as being above the 99th or below the 1st percentile for the given feature, were deleted and marked as missing. The algorithm is capable of making predictions in the presence of missing data. When calculating the tabular scores for the comparators, missing values added 0 points towards the total score.

### Machine Learning Model

The machine learning mortality predictor was developed using XGBoost [28], a gradient boosting technique. Gradient boosting combines results from multiple decision trees, where each decision tree divides patients into successively smaller groups based on their vital sign values. For example, one branch of a decision tree might divide patients into two groups depending on if their heart rate was over or under 90 beats per minute. Each tree ends in a set of leaves, where each patient is represented in a single leaf based on their set of measurements. The particular leaf to which the patient is sent on each decision tree yields a risk score. The score from each tree is then weighted and totaled to give the model’s final prediction for the specified patient. A variety of parameter combinations controlling tree depth and maximum weights assigned to each leaf were used to identify the best performing model.

To train the model to make mortality predictions without discrimination, we preprocessed our training data in two steps. These steps were performed with the intention of removing aspects of the data that reflect systemic inequities in health across racial groups while maintaining the aspects of the data that reflected relevant patient measurements and outcomes. First, the patients were separated into age groups defined as younger than 18 years, 18 to 29 years, 30 to 39 years, 40 to 49 years, 50 to 59 years, 60 to 69 years, and 70 years and older. This was to control for the high correlation between age and mortality rate. Second, individual training examples were given weights based on mortality status and race within each age strata using a reweighting scheme. This was done by weighting each training example in the following way: first the expected probability of observing the given combination of race and mortality was calculated by assuming statistical independence of these variables. This was then compared with the observed probability of the variable combination found in the training data. This ratio of expected to observed probability was then used as the weight for each training example. This ratio can be considered a demographic prevalence ratio and is based on the method originally described by Kamarin and Calders [7]. Example code for this preprocessing method is included in Multimedia Appendix 1.

To train and test the machine learning algorithm, we used 10-fold cross-validation. Reported performance metrics are an average of each model’s performance on each of the 10 test sets. Several baseline models were assessed as candidates for development. We compared the performance of gradient-boosted trees (using XGBoost), logistic regression, and multilayer perceptron models for mortality prediction. We found that gradient-boosted trees performed best at baseline and chose them as the primary model type on which to perform all subsequent experiments. Pairwise comparisons between gradient-boosted trees and alternative model types without preprocessing were made using a Student *t* test for area under the receiver operating characteristic (AUROC) and the McNemar test for distinguishing predictions.

### Statistical Analysis

The predictive performance of all comparators was assessed by associating each comparator score with the mortality rate found in training encounters that had the same score. In addition, the highest probability observed for a score was carried forward to the next score value if it was found to have a lower probability of death to ensure increasing scores were monotonically associated with an increased probability of the outcome. Comparator scores were assessed on each of the 10 folds used in cross-fold model validation.

For all models, predictions were made after 24 hours of ICU data were collected, with the mortality outcome defined as any in-hospital mortality at end of stay. Overall predictive performance of comparators and the machine learning algorithm are reported using the area under the receiver operating characteristic, sensitivity, specificity, diagnostic odds ratio (DOR), and positive and negative likelihood ratios (LR+ and LR–).

To assess whether the machine learning algorithm and each comparator identified similar at-risk individuals, the McNemar test was used, comparing performance of the two systems at a sensitivity around 0.75. Performance was assessed both on the overall sample and after stratifying by race. Racial categories were defined as white and nonwhite, where only non-Hispanic white patients were included in the white category (eg, a white Hispanic patient was considered nonwhite for the purpose of this analysis).

Model bias was assessed using the equal opportunity difference statistic. Equal opportunity difference measures the distribution of false negative results across two groups produced by each prediction method and assesses the difference in the false negative rate between the groups. False negative results are of particular importance for mortality prediction tools as a failure to provide an alert for a patient at risk of mortality may lead to a lack of timely care and an increased risk of death. Under an unbiased predictor, the false negative rate should not differ between the racial groups; the expected value of the equal opportunity difference statistic for an unbiased predictor is therefore 0. Significance of the equal opportunity difference statistic was assessed using a Student *t* test under the null hypothesis that the equal opportunity difference was equal to 0. Equal opportunity difference statistics were assessed separately for all prediction models. For all statistical tests, an alpha of .05 was used.

The final XGBoost model, trained on preprocessed data as described above, was compared with the XGBoost model trained on unpreprocessed data to assess the impact of the preprocessing techniques. Finally, we also assessed feature importance using Shapley values for machine learning models developed with and without preprocessing of the training data to assess the impact of the preprocessing procedure. We additionally compared feature importance for the final machine learning model across white and nonwhite racial groups.

## Results

Patient demographic data from the MIMIC-III [22] database for the full cohort and after stratifying by race are presented in Table 1. A total of 28,460 patients were included in the final study sample, 23,263 (81.74%) of whom were white and 5197 (18.26%) of whom were nonwhite.

Several models were considered for predicting mortality. When compared with logistic regression and multilayer perceptron classification methods for their mortality prediction performance, the XGBoost model exhibited improved prediction performance as measured by AUROC, sensitivity, specificity, DOR, and LR+/– (Multimedia Appendix 1). Comparisons between XGBoost and other classification models were statistically significant (*P*<.001).

The final XGBoost model was trained to be unbiased by preprocessing the training data to ensure statistical equivalence of false negative rates for both white and nonwhite patient populations. The model outperformed all rules-based comparator scoring systems in predicting in-hospital mortality, achieving an AUROC of 0.78. The algorithm demonstrated improved sensitivity, specificity, DOR, and LR+/– as compared with comparator scores (Table 2). All pairwise comparisons between the algorithm and a rules-based comparator were statistically significant (*P*<.001, by McNemar test). Performance results for the machine learning algorithm on white and nonwhite patient populations are included in Multimedia Appendix 1.

**Table 1:** Demographic and medical information history for the Medical Information Mart for Intensive Care–III study sample by discharge status.

Characteristic	Full sample		White subset		Nonwhite subset	
	Living (n=19,269)	Deceased (n=9191)	Living (n=15,394)	Deceased (n=7896)	Living (n=3875)	Deceased (n=1322)
Female, n (%)	8129 (42.19)	4269 (46.45)	6313 (41.01)	3672 (46.66)	1816 (46.86)	597 (45.16)
Age, mean (SD)	60.11 (17.4)	71.31 (14.7)	61.4 (17.1)	71.91 (14.4)	54.99 (17.7)	67.73 (15.5)
Cardiovascular, n (%)	15,869 (82.36)	8085 (87.97)	12,790 (83.08)	6928 (88.04)	933 (24.08)	394 (29.80)
Renal, n (%)	5778 (29.99)	3867 (42.07)	4376 (28.43)	3243 (41.21)	391 (10.09)	201 (15.20)
Diabetes, types 1 and 2, n (%)	3843 (19.94)	1854 (20.17)	2903 (18.86)	1517 (19.28)	3079 (79.46)	1157 (87.52)
COPD^a^, n (%)	1626 (8.44)	1139 (12.39)	1428 (9.28)	1032 (13.11)	1402 (36.18)	624 (47.20)
Sepsis, n (%)	729 (3.78)	321 (3.49)	534 (3.47)	269 (3.42)	195 (5.03)	52 (3.93)
Severe sepsis, n (%)	3877 (20.12)	2517 (27.39)	2944 (19.12)	2123 (26.98)	712 (18.37)	322 (24.36)
Septic shock, n (%)	1823 (9.46)	1271 (13.83)	1432 (9.30)	1070 (13.60)	401 (10.35)	184 (13.92)
Mental health disorder, n (%)	7351 (38.15)	2994 (32.58)	5882 (38.21)	2563 (32.57)	1469 (37.91)	431 (32.60)
Pneumonia, n (%)	3265 (16.94)	2186 (23.78)	2553 (16.58)	1864 (23.69)	198 (5.11)	107 (8.09)
Liver^b^, n (%)	1602 (8.31)	1020 (11.10)	1201 (7.80)	836 (10.62)	940 (24.26)	337 (25.49)
Cancer, n (%)	2941 (15.26)	2766 (30.09)	2297 (14.92)	2335 (29.67)	644 (16.62)	431 (32.60)
HIV/AIDS, n (%)	201 (1.04)	102 (1.11)	115 (0.75)	62 (0.79)	86 (2.22)	40 (3.03)

^a^COPD: chronic obstructive pulmonary disease.

^b^Acute and subacute necrosis of liver, chronic liver disease and cirrhosis, liver abscess and sequelae of chronic liver disease, and other disorders of liver.

The algorithm was found to be unbiased as measured by the equal opportunity difference score, with an insignificant *P* value for model bias and an equal opportunity difference of 0.016 (*P*=.20). The APACHE score was also found to be unbiased, with an equal opportunity difference of 0.019 (*P*=.17). However, both SAPS II and MEWS were found to have statistically significant bias as measured by equal opportunity difference, with equal opportunity difference values of 0.038 and 0.074 and *P* values of .006 and <.001, respectively.

**Figure 2:**
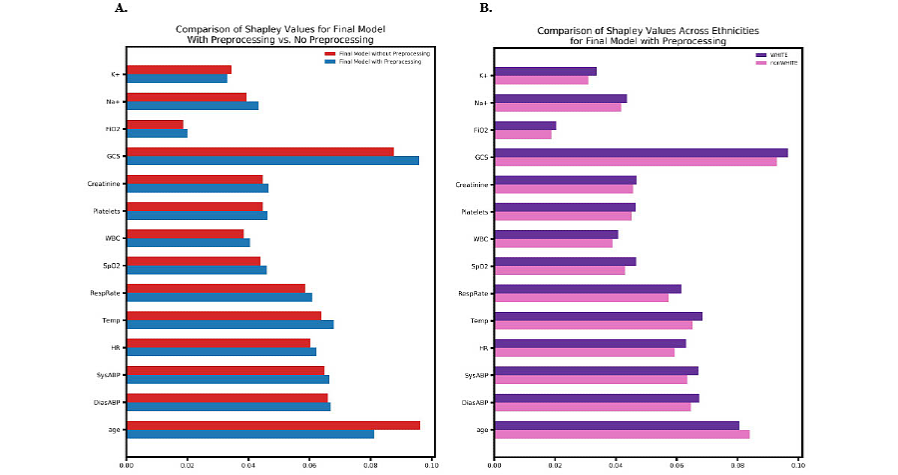
Comparison of feature importance between (A) models trained with and without preprocessing of the training data and (B) white and nonwhite subgroups on the model trained with preprocessing of the training data.

Preprocessing of the training data was found to make a meaningful difference in model performance. On an XGBoost model trained on unpreprocessed data, the equal opportunity difference was found to be larger, at 0.023 (*P*=.07). A full comparison of models trained with and without data processing are presented in Multimedia Appendix 1. In assessing feature importance for models trained with and without preprocessing of the training data, we found differences in the importance of age and Glasgow coma scale features (Figure 2A), which may reflect differences in the distribution of age and life expectancy across race in the general population and differences in disease severity upon presentation to the ICU across racial groups. In particular, nonwhite patients were generally found to be younger than white patients before preprocessing, indicating an interaction between age and race on mortality outcome prediction. After preprocessing of the training data, feature importance was found to be similar for all measured features across racial groups (Figure 2B).

**Table 2:** Performance metrics for the machine learning algorithm and all comparator scores for mortality prediction on the total study population.

Statistics	MLA^a^	MEWS^b^	APACHE^c^	SAPS-II^d^
AUROC^e^	0.780	0.580	0.700	0.660
Sensitivity	0.751	0.523	0.678	0.674
Specificity	0.656	0.577	0.596	0.511
DOR^f^	5.739	1.499	3.106	2.157
LR+^g^	2.181	1.238	1.678	1.378
LR–^h^	0.380	0.826	0.540	0.639

^a^MLA: machine learning algorithm.

^b^MEWS: Modified Early Warning Score.

^c^APACHE: Acute Physiologic Assessment and Chronic Health Evaluation.

^d^SAPS II: Simplified Acute Physiology Score II.

^e^AUROC: area under the receiver operating characteristic.

^f^DOR: diagnostic odds ratio.

^g^LR+: positive likelihood ratio.

^h^LR–: negative likelihood ratio.

## Discussion

### Principal Findings

In this study, we examined whether a machine learning algorithm is capable of predicting mortality with reduced racial bias as compared with commonly used early warning and severity scoring systems. We found evidence of statistically significant bias as measured by the equal opportunity difference measures of MEWS and SAPS II, but no evidence of bias for the machine learning algorithm or for APACHE. In addition, the algorithm displayed better overall performance as measured by AUROC, sensitivity, specificity, DOR, and LR+/–. The combination of superior predictive performance and unbiased performance indicate that the machine learning algorithm may be more appropriate than any of the comparator scores for risk stratification in clinical settings as the algorithm appears most capable of accurately identifying all patients at risk of in-hospital mortality.

The ability to demonstrate that a risk prediction tool can be used without inherent racial bias is a crucial step toward minimizing health care disparities. Large, well-designed cohort studies have found significant evidence of racial bias in commonly used scoring systems, including reduced accuracy of MEWS when implemented on an Asian population [23] and consistently lower acuity scores for nonwhite patients when examining the performance of the ESI [20-22]. This body of evidence indicates that nonwhite patients may be subject to inferior health care. Importantly, these persistent racial disparities in the provision of health care may be reflective of systematic failure to identify minority patients most likely to require immediate or aggressive care.

Continued research on ways to deliver equitable performance from systems such as MEWS and the ESI is essential. However, while machine learning algorithms can be subject to racial bias in their own right [7,9], well-designed algorithms may offer advantages over traditional scoring systems. These advantages are only present, however, if the algorithm is intentionally designed with the aim of minimizing racial bias. This paper has demonstrated the success of a preprocessing technique [7] that has benefits of making minimal alterations to the training data and not requiring costly alterations to the model training procedure. When machine learning prediction models are developed without this or a similar technique to counteract racial bias, algorithms used within the health system have been found to be less accurate for racial minorities. Obermeyer and colleagues [8] found that an algorithm commonly used across the United States had reduced accuracy for nonwhite patients due to the use of health care costs standing as a proxy for overall patient risk in the model output. Further, they found that minority patients generally had higher comorbidity index values when compared with white patients with the same overall risk score, indicating a systematic underestimation of the health care needs of nonwhite patients. Obermeyer and colleagues [8] found that reframing the model prediction task (in this case, from predicted costs to a measure of predicted health) minimized racial bias in model accuracy. A study by Chen et al [9] similarly found that machine learning algorithms displayed higher error rates when predicting psychiatric readmission and mortality in minority patients as compared with white patients.

This research seeks to fill a gap beyond addressing bias that can occur with clinical diagnostic testing. In addition, it adds to the body of evidence regarding how systemic health care inequalities emerge and persist and shows that poor calibration of traditional prediction scores as pertains to nonwhite populations can potentially influence health care decision making in the United States [29,30]. Although more research is needed to assess bias and disparities across a wide range of settings and applications, the potential harm that can come from bias in simple severity scores is made clear by recent recommendations surrounding COVID-19. Several recommendations for providing care and allocating limited resources have suggested that aggressive treatment be provided to patients based on assessment by MEWS, SAPS II, APACHE, or similar severity scores [31-33]. However, bias in severity scores used to triage COVID-19 patients could widen existing racial disparities in COVID-19 [34], and this work makes clear that less biased methods are achievable and preferable for such uses.

### Limitations

This study has several important limitations. First, the study used retrospective patient medical records. There are known inaccuracies in the way that race and ethnicity are recorded in medical records; this in turn may have impacted the accuracy of our results [35]. Additionally, our analysis compared nonwhite to white patients and did not consider more nuanced categories of racial identity. There may be nuances in the accuracy of the algorithm and its comparators across these groups. We also note that overall, our study sample was largely white, with only around 18% of our sample reporting nonwhite race or ethnicity. The predominance of white patients in this study may have biased results; validation of this model on additional datasets is warranted. Research has indicated the potential for bias in the way that seemingly objective measures such as heart rate, respiratory rate, and spirometry, as well as pain assessments, are made across racial groups [22,36-38]. Lab measurements also pose the potential for bias due to the incorporation of race corrections in measures such as estimated glomerular filtration rate. Additionally, there are further ways of measuring and assessing discriminatory predictive performance not assessed in this paper. This is a retrospective study, and we therefore cannot determine the impact this algorithm will have on patient care in a live health care setting.

### Conclusions

We believe that the potential for bias through this mechanism is mitigated in our machine learning method as compared with rules-based methods. This is due to our incorporation of several laboratory measures collected using standardized methods not incorporating race corrections, use of measurements obtained at a variety of time points and therefore likely assessed by a variety of clinicians, and statistical methods used to minimize bias during the model training process. Despite its limitations, the algorithm examined in this study shows promise as one of many necessary steps toward decreasing racial disparities in health care.

## Appendix

Multimedia Appendix 1 Supplementary materials.

## Abbreviations

AI: artificial intelligence
APACHE: Acute Physiologic Assessment and Chronic Health Evaluation
AUROC: area under the receiver operating characteristic
DOR: diagnostic odds ratio
ESI: emergency severity index
ICU: intensive care unit
LR+: positive likelihood ratio
LR–: negative likelihood ratio
MEWS: Modified Early Warning Score
MIMIC-III: Medical Information Mart for Intensive Care–III
SAPS II: Simplified Acute Physiology Score II
