# Supplementary material for: A Racially Unbiased, Machine Learning Approach to Prediction of Mortality: Algorithm Development Study
Source: JMIR Public Health Surveill. 2020 Oct 22;6(4):e22400. doi: 10.2196/22400 (PMC7644374; doi:10.2196/22400)
Supplement: Multimedia Appendix 1 [file publichealth_v6i4e22400_app1.docx]

**Multimedia Appendix 1. Supplementary materials.**

**Supplementary Table 1.** Comparison of predictive performance for logistic regression, multilayer perceptron, and XGBoost methods of in-hospital mortality prediction without preprocessing of the training data. AUROC is reported as mean (standard deviation).

|  | Logistic Regression | Multilayer Perceptron | XGBoost |
| --- | --- | --- | --- |
| AUROC | 0.749  (0.004) | 0.759  (0.006) | 0.779  (0.006) |
| Sensitivity | 0.75 | 0.748 | 0.747 |
| Specificity | 0.616 | 0.632 | 0.665 |
| Accuracy | 0.659 | 0.669 | 0.692 |
| DOR | 4.811 | 5.092 | 5.872 |
| LR+ | 1.954 | 2.031 | 2.23 |
| LR- | 0.406 | 0.399 | 0.38 |

Abbreviations used: AUROC: area under the receiver operating characteristic. DOR: diagnostic odds ratio. LR: likelihood ratio.

**Supplementary Table 2.** Comparison of XGBoost performance on non-white and white patient populations. AUROC is reported as mean (standard deviation). Metrics are reported at ≤0.75 sensitivity.

|  | Non-white | White |
| --- | --- | --- |
| AUROC | 0.785 (0.024) | 0.772 (0.007) |
| Sensitivity | 0.737 | 0.753 |
| Specificity | 0.681 | 0.65 |
| Accuracy | 0.695 | 0.685 |
| NPV | 0.883 | 0.837 |
| PPV | 0.44 | 0.523 |
| DOR | 5.962 | 5.654 |
| LR+ | 2.306 | 2.149 |
| LR- | 0.387 | 0.38 |
| F1 | 0.551 | 0.618 |

**Supplementary Table 3.** Comparison of XGBoost performance with and without preprocessing of the training data. AUROC is reported as mean (standard deviation).

|  | XGBoost without Preprocessing | XGBoost with preprocessing |
| --- | --- | --- |
| AUROC | 0.779 (0.006) | 0.775 (0.007) |
| Sensitivity | 0.747 | 0.751 |
| Specificity | 0.665 | 0.656 |
| DOR | 5.872 | 5.739 |
| LR+ | 2.23 | 2.181 |
| LR- | 0.38 | 0.38 |
| Equal Opportunity Difference | 0.023 | 0.016 |
| Equal Opportunity Difference p-value | 0.074 | 0.204 |

Abbreviations used: AUROC: area under the receiver operating characteristic. DOR: diagnostic odds ratio. LR: likelihood ratio.

**Pseudocode:**

*Our version of the reweighting algorithm, returns* Xnew, Anew, Ynew, Wnew*:*

- *Inputs:* quantitative data X, ethnicity labels A, endpoint labels Y
- *Start:*
  - Create empty lists Xnew, Anew, Ynew, Wnew
  - *For each age group:*
    - Take the patients in the training data that fall in this age group and place their quantitative data, ethnicity labels, and endpoint labels into lists, with patients identifiable by matching the index in each list:
      - Xage=x1, x2, ..., xN(age)
      - Aage=a1, a2, ..., aN(age)
      - Yage=y1, y2, ..., yN(age)
    - Define the following function which will be used to compute the sample training weights, where y is the endpoint value and a is the ethnicity label:
      - w(y,a)=xi Xage : yi=y1xi Xage : ai=a1xi Xage : yi=y, ai=a1
    - Define a list of training weights for the patients in this age group to be the following:
      - Wage=w(y1,a1), w(y2,a2), ..., w(yN(age),aN(age))
    - Append entries in Xage, Aage, Yage, Wage to Xnew, Anew, Ynew, Wnew, respectively
  - Shuffle Xnew, Anew, Ynew, Wnew
  - Use Xnew, Ynew as inputs and labels, with sample weights Wnew, when training the learning algorithm
- *End*
